# New frontiers review of some recent conservation techniques of organic and inorganic archaeological artefacts against microbial deterioration

**DOI:** 10.3389/fmicb.2023.1146582

**Published:** 2023-03-16

**Authors:** Neveen S. Geweely

**Affiliations:** Department of Botany and Microbiology, Faculty of Science, Cairo University, Giza, Egypt

**Keywords:** inorganic archaeological objects, Organic artifacts, microbial deterioration, fungi, bacteria, new conservation, antimicrobial, biocides

## Abstract

The information on the advances and technology of some recent conservation methods (2020–2023) of organic and inorganic archaeological objects against microbial deterioration is recorded. An outline of comparative new protective methods for conserving plant-origin organic artefacts {Fibers (manuscripts, textile) and wood}, animal-origin organic artefacts (painting, parchment and mummies) and inorganic stone artefacts were investigated. The work not only contributes to the development of safe revolutionary ways for more efficient safe conservation of items of historical and cultural worth but also serves as a significant diagnostic signature for detecting the sorts of microbial identification and incidents in antiques. Biological technologies (environmentally friendly green biocides) are the most used recent, efficient and safe strategy acceptable as alternatives to stop microbial deterioration and prevent any potential interactions between the biological agent and the artefacts. Also, a synergistic effect of combining natural biocides with mechanical cleaning or chemical treatments was suggested. The recommended exploration techniques should be considered for future applications.

## 1. Introduction

Cultural heritage serves as a reflection of human identity and provides documentation of historical existence and activity. The biological systems and metabolic processes of microorganisms that contribute to biodeterioration can cause the loss and weakening of original materials and structures as well as the discolouration, deformation and loss of ethnographic deposits (Romero et al., 2021). In order to preserve our cultural heritage for future generations, interdisciplinary research teams embraced by microbiologists, geologists, chemists, physicians, archeologists, and ecologists should be involved in the challenging research opportunities of heritage conservation science. The mechanisms of biodeterioration by microbial communities lead to the right choice of efficient conservation technique (Bracci et al., 2020).

The use of molecular and chemical tools (mass spectrometry and next-generation sequencing) in the field of heritage conservation has highlighted the intriguing potential of a variety of diverse nanomaterials (Beata, 2020). Antimicrobial Activities of novel chemical molecules were evaluated by Geweely (2009a, 2011), Ghozlan et al. (2015), Salem et al. (2022), and Kamel et al. (2022). Several pathological states of soft tissues and internal organs made it possible for researchers to determine the origin of antiques, the course of development and the appearance of a wide range of infections in historical contexts (Shin and Bianucci, 2021). Metagenomics, transcriptomics, metabolomics and proteomics are systemic techniques referred to as omics methods which used to investigate the biodeterioration of cultural stuffs (Gutarowska, 2020). According to the materials used and the manufacturing processes, artifact materials may generally categorize using common typologies. Organic artifacts make up a significant amount of the items now shown in museums across the world, thought to be particularly susceptible to deterioration since microbial attacks can cause their destruction with different effects (Fabbri and Bonora, 2021). The primary difference between the rate at which inorganic artifacts deteriorate and the rate at which organic stuff degrades is how rapidly organic matter degrades (Bauer et al., 2021). According to Gioventù et al. (2020) who stated that bacteria play a significant role in the mineralization processes and the breakdown of complex organic molecules (carbohydrates, proteins, lipids, and cellulose) that lead to the completion of the organic matter cycle. A typical museum setting has a wide variety of microbial species found in nourishing environments that have a special affinity for glue, starch, cellulose and protein-rich materials (fur, wool, silk and skins) as reported by Bangstad (2022).

The traditional chemical and mechanical treatments generate unsatisfactory results or have short-term impacts. The researchers looking into the world of natural chemicals (plant extracts, green biocides or bio-based belongings) as alternatives that will be more readily biodegradable and environmentally non-threatening techniques safe for human health and the environment for removing biological patinas. Numerous of these green biocides are blends of polypeptides produced by plants, including phenols, polyphenols, terpenoids, essential oils, alkaloids and lectins (Cremonesia and Casoli, 2021). Also, El-Shahawy et al. (2010) directed toward the study of new antimicrobial drugs due to the misuse of antibiotics and the emergence of multi-drug resistant microbial strains, along with their lack of efficacy and adverse effects. According to Palla et al. (2020) who recorded that the utilization of blue and green bioactive compounds (bio-surfactants, chelating agents, natural biocides and essential oils) isolated from microorganisms, plants and marine creatures is a unique feature of organic cultural heritage conservation biotechnologies. Traditional biocides are still widely used to counter biodeterioration although their toxicity (Villa et al., 2020).

Controlling the microbiological degradation of cultural assets is related to the fact that various methods for determining treatment efficacy are frequently subjective and non-replicable (ICOMOS, 2020). A complex interaction between ambient temperature and humidity is crucial to the *in situ* conditions and variabilities of the water available on the surface and inside the cultural heritage, so to improve cultural heritage conservation research, more precise measurements at the sampling location and the time of the sampling are required (Chen and Gu, 2022). The effectiveness of several control techniques on the same items is crucial for environmental safety (Mascalchi et al., 2020). It is possible to lower the populations of potentially pathogenic heterotrophic bacteria that produce acid by installing air-purifying systems in exhibition rooms, such as photocatalytic ionizers and microclimate-controlled settings, which are increased by a variety of anthropogenic activities. The conservation of ancient artifacts against microbial decay faces several difficulties, so every effort should be taken to preserve historic materials and transmit them to future generations (Geweely, 2022). The effectiveness of new technologies and practices should be enriched to develop the environment for both people and cultural heritage items (Katsivelal et al., 2021). As a result of technological advancements, more techniques and their combination necessary to remove unwanted microorganisms have opened up new opportunities for both microbiologists and conservators (Rusu et al., 2020).

The current study aims to propose a new informative study to aid the researchers in considerate recent, efficient and safe preservation techniques for the conservation of archaeological organic and inorganic objects against microbial deterioration as well as their benefits and drawbacks on artifacts, the environment and human health in addition to future perspectives in this field.

### 1.1. Archaeological deteriorating microbes

Microorganisms have a crucial role in both natural and anthropogenic processes that lead to the destruction of the world’s cultural heritage. In-depth insights into biodeterioration may be possible through a combination of physicochemical studies, and culture-dependent and culture-independent characterization of bacteria and fungi involved in the destruction of various historical materials (Pyzik et al., 2021). A crucial understanding of the active biodeterioration processes, the efficacy of conservation techniques for the protection of the world’s cultural heritage, the entire microbial community composition and the active microorganisms recorded from genomic DNA and RNA were needed (Ding et al., 2020). Also, Meng et al. (2020) stated that to give substantial information for the preservation of the monument, the active microbial flora of the community must be investigated using RNA rather than DNA. According to Gutarowska (2020) who reported that repeated biocidal treatments lead to resistance in the target biological agents leading to change in the biofilm structures and encouraging the formation of more threatening biodeteriogens, so omics methods used to analyze the biodeterioration of cultural properties. It is crucial to create systematic and accurate microbial monitoring procedures for the protection of delicate ancient objects from microbial degradation and assemble significant information about the practices and habits of a particular historical era. The investigation of the deteriorating microbiota was made by the combination of non-invasive sampling, conservative microbiological techniques, molecular methodologies, high-throughput sequencing, DNA analysis, determining the diversity of cultivable bacterial populations and the choice of an adequate isolation medium (Kisová et al., 2020).

The significance of biofilms on historic surfaces has been examined in recent conservation literature, taking into consideration both biodeterioration and bio-protection mechanisms (Favero-Longo and Viles, 2020). The detection of bacterial species especially human diseases bacteria would be made possible by a combination of two types of media using meta-barcoding studies to examine the bacterial contamination of the museum’s air which helps to confirm the biochemical and eco-physiological functions of microorganisms in degradation (Dziurzynski et al., 2020). New analysis insights into microorganisms and their metabolisms was applied for the protection of artworks and conservation in terms of cost, effectiveness, safety and environmental sustainability (Favero-Longo and Viles, 2020). The examination of the status of conservation and treatments of monuments and cultural heritage items has expanded by new analytical techniques like high-throughput next-generation sequencing (De Leo and Jurado, 2021). The key elements controlling the growth of bacteria and fungus on organic artifacts have been identified as a possible use for nanomaterials in the preservation of organic cultural heritage objects. Microbial species frequently have an impact on a variety of artifact kinds of *Ascomycetes*, *Aspergillus*, *Paecilomyces*, *Penicillium*, *Cladosporium*, *Eurotium Chrysosporium*, *Chaetomium*, *Monoascus*, *Epicoccum*, *Trichoderma* and *Stachybotrys* that penetrating through destruction or enzymatic action, causing staining of the artifact (the foxing phenomenon) or result in the appearance of traces or hazardous substances (Fistos et al., 2022).

The prevention of fungal contamination of interior objects and indoor air directly related to visitors’ and employees’ health, requires careful microclimate management within storage or display halls, together with microbiological monitoring, green bioremediation and cleaning procedures (Ilies et al., 2022). The visual examination was used to assess the fungal degradation elements of historical artifacts, including color change, brittleness, weakness and erosion. Four hydrolytic enzymes (Cellulase, amylase, gelatinase and pectinase) that are highly active and essential for biodegradation and breakdown by deteriorating fungal strains. Additionally, employees must receive training on how to get rid of deteriorating spurs (dust and mold) (Abdel-Maksoud et al., 2022). Borrego et al. (2021a) recorded that the prolonged and severe contact with biological agents, the mycological quality of the interior environment leading to the development of allergies and other disorders that harm the staff’s health and cause the biodeterioration of documents. The identification of fungi in the nasal mucosa of the archive employees was noted, were *Aspergillus*, *Cladosporium*, and *Penicillium* were the most prevalent genera. The skin of 40.3% of the staff members responded well to one or more fungus extracts. Asthma was identified as occurring in 54.2% of the employees. Worker exposure to the more or less polluted atmosphere of the archive for nine to 12 years promotes the colonization of fungal species in the nose, which can cause the onset or worsen of allergy conditions such as asthma and rhinitis. Wei et al. (2022) found that *Firmicutes*, *Actinobacteria*, *Actinobacteria*, *Proteobacteria*, *Acidobacteriota*, *Methylomirabilota*, *Chloroflexi* and *Bacteroidota* were the eight most prevalent bacterial species found in soil samples from tombs. Also, the excretion of pigments and organic acids was used to identify the pathogenic and biodeteriogenic characteristics of live fungi (*Aspergillus*, *Cladosporium*, and *Penicillium*) found in interior settings of air-conditioned repositories, where 100% of the isolated spores analyzed can enter the upper respiratory system, while only 71.7% of the isolated *Aspergillus*, *Penicillium*, *Cladosporium sphaerospermum* and *Acrodontium simplex* spores can enter the pulmonary alveoli (Borrego et al., 2021b). The fungus which has strong biodegradation abilities and high enzyme capacity was *Aspergillus*, which poses a threat to the preservation of cultural heritage (Romero et al., 2021).

Recent developments have revealed that some microbes and microbial-based technologies may play a role in the preservation of cultural heritage and offer valuable benefits (Bauer et al., 2021). Fourteen and eleven biodeteriogenic species were isolated from the indoor air and dust, respectively, while the majority of the genera were *Aspergillus*, *Cladosporium* and *Penicillium*, demonstrating the possible risk of the fungal environments for the protected recorded history (Borrego et al., 2022a). The staff’s health was evaluated concerning the airborne fungal contamination of naturally ventilated repositories in historical archives. The fungal concentrations ranged from 135.6 CFU/m^3^ to 421.1 CFU/m^3^, demonstrating a fluctuating environmental quality over time. *Cladosporium* and *Aspergillus* were the two most common genera. *A. flavus* predominated in indoor air, *A. niger* and *C. cladosporioides* most closely resembled outside air. The earliest discoveries for the surroundings of archives were *Coremiella, Talaromyces*, *Aspergillus uvarum*, *Alternaria ricini*, and *Cladosporium staurophorum*, where xerophilic species (*A. flavus*, *A. niger*, *A. ochraceus*, and *A. ustus*) indicators of the presence of moisture issues in the repositories. They are also opportunistic pathogens and toxigenic species, where their concentrations were higher than the recommended, demonstrating the potential risk to which the archive staff was exposed in a circumstantial way (Borrego et al., 2022b).

## 2. Conservation of plant origin organic deteriorated archaeological objects

### 2.1. Fibers (manuscripts, textile)

Artefacts made of paper have unique characteristics that make them more vulnerable to deterioration. These characteristics include their physical characteristics as well as the presence of ink and pigment on their surface. Each of these elements has the potential to accelerate the microbial deterioration of paper artifacts, making the development of remediation techniques an essential area of research (Abdel-Kareem et al., 2022). A historical manuscript’s degrading features show high efficiency in the fungal production of hydrolytic enzymes including cellulase, amylase, gelatinase and pectinase, which are crucial in biodeterioration (Fouda et al., 2022). Archaeological records should be preserved carefully for future generations as they are seen to be a valuable resource for a deeper understanding of our culture and heritage (Buragohain et al., 2022).

Microorganisms destroy all fabrics by reducing their tensile strength and flexibility (Hussien, 2022). Buragohain and Kumar (2021), Roberto et al. (2021), and Somarajan (2021) preserved texts from the digital period. The greatest amount of fungal contamination found in textiles was *Aspergillus* sp. contamination which should be highlighted as one sign of potential environmental problems. In two of the textile settings, *A. fumigati* and *A. circumdati* were particularly significant. Electrostatic dust cloths was used as a sampling technique for conservation and restoration and the exposure to air pollutants is reduced and effective cleaning procedures may make the working environment safe (Viegas et al., 2022). The irradiation parameters for the highly resistant colonizer (*Cladosporium sphaerospermum*) and the naturally existing microbiota collected on paper samples were examined *In vitro*. Gamma irradiation is a well-known conservation technique and a decrease in the microbiota was seen with a radiation dosage of 7 kGy, leading to consideration of the 8.2 kGy recommended dose (Marušic et al., 2020).

**Figure 1:**
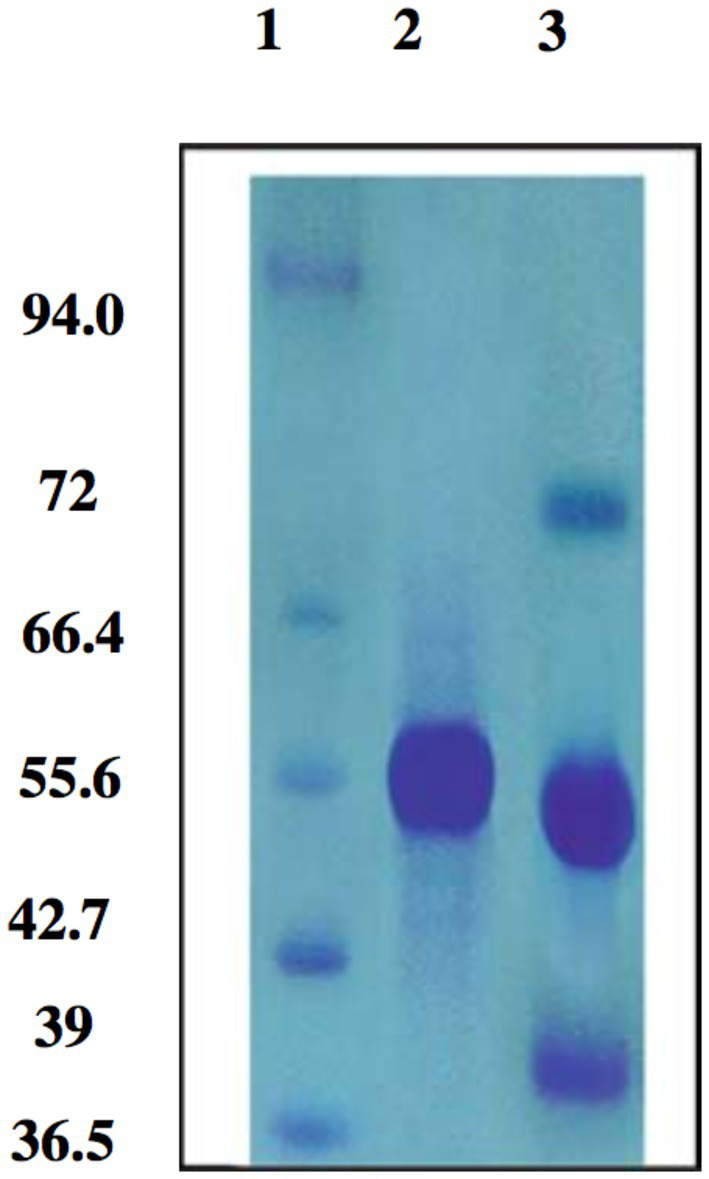
SDS-PAGE of *Candida glabrata* cell wall protein. Marker proteins are located in Lane 1, One pure band of 55.6 KDa in *C. glabrata* untreated zinc protein in Lane 2 and Zinc treated *C. glabrata* protein showing appearance of two new protein bands with molecular weights of 72 and 39 KDa in Lane 3 (Geweely, 2009b).

Zinc oxide nanoparticles can provide benefits like self-cleaning capabilities and protection for archaeological papers with no discernible difference in the tensile strength of the paper treated with the nanoparticles (Franco-Castillo et al., 2021). Fouda et al. (2021) documented the effectiveness of nanocomposites of silver nitrate as a coating agent to guard paper against highly degrading microbes, including disruption of the cell wall with plasma membrane, inhibition of protein synthesis and DNA replication and the enhanced oxidation of cell components as reported by Omanovic-Miklicanin et al. (2020). The main target of the Zn(II) metal is the microbial cell wall, which is crucial for growth and zinc metal can quickly change the microbe morphology as shown in Figure 1. The use of inorganic nanoparticles for the preservation and preventive treatments of paper artifacts was recorded by Fistos et al. (2022). A traditional blouse fabric from Romania, dating back 100 years was cleaned and preserved using a combination of conventional and cutting-edge techniques. The degree of preservation of textile objects is affected by the antibacterial effects of natural wood ash (lye) and silver nanosuspensions at 30 and 70 ppm on the material of the blouse. It is environmentally non-threatening and does not harm the fabrics’ base materials. Lye and silver nanoparticles both have antibacterial/fungal capabilities, which led to the discovery that bacterial colonies were decreased by more than 95%, and the effects would last for a considerable amount of time (Ilies et al., 2022).

The extract of *Acacia nilotica* was used at a concentration of less than 5% to treat organically infected archaeological documents that date back to 1,300 AH and 1882 AD. The microscope was used to verify that the activities of bacteria and fungi had stopped (Hosnil et al., 2021). Ilies et al. (2021) stated that the antimicrobial effects of essential oils and plant extracts have in the short term must be tested in the future to ensure the enhanced preservation of historical textiles and the health integrity of the restorers and visitors who view them in museums, collections or exhibitions. The essential oils of lemon (*Citrus limon*), mint (*Mentha piperita*) and lavender (*Lavandula angustifolia*) proved to be inexpensive, simple and non-destructive solutions for cleaning and prevention the spreading of bacteria and mold spores as a great alternative to the conventional chemical treatments used to preserve cultural heritage artifacts that are afflicted with six distinct species of yeast (*Candida guilliermondii*, *C. sphaerica*, *Cryptococcus albidus*, *C. laurentii*, *C. neoformans* and *Sporobolomyces salmonicolor*), six different types of mold (*Penicillium* sp., *Aspergillus* sp., *Trichoderma* sp., *Cladosporium* sp., *Stachybothris* sp. and *Botrytis* sp.) and bacteria (*Staphylococcus* sp.) that have been found in the museum’s air and have been shown to damage fabrics. The essential oils manner a potential risk to human health, particularly for children and people’s health, particularly in the case of kids and people who have allergic rhinitis or other allergic respiratory conditions. *Aspergillus* and *Staphylococcus* have been demonstrated to be inhibited by lavender oil, whereas *Cladosporium* and *Botrytis* were inhibited by the mint essential oil. Essential oils can be thought as fabric fungal decontamination treatments that help to preserve the clothing on display in museums.

Additionally, an ancient Egyptian textile from King Khufu’s time was examined and suffered from a serious fungal infection caused by *Aspergillus flavus*, *Aspergillus midulans*, *Aspergillus niger*, *Paecilomyces variotii*, *Penicillium* sp. Gram-negative bacilli, Gram-negative cocci and Gram-negative bacilli. Cedar oil, a naturally occurring ingredient, was effectively utilized to sterilize textiles against deteriorating fungi by pouring cedar oil within a tightly closed room housing of the artifact for 2 weeks. The sterilizing process was finished inside the closed room, which was constructed of polyethene (Nabil et al., 2021).

### 2.2. Wood

The micro-morphological patterns created during microbial degradation of lignified cell walls of buried and waterlogged archaeological woods is a crucial diagnostic signature for identifying the types of microbial attacks present in woods and help in the development of targeted methods for more effective preservation of wooden objects of historical and cultural importance. Also, the identification of ancient wood species and description of their weathering processes are essential first stages in the scientific preservation of wooden cultural heritage (Singh et al., 2022).

Identification of the wood species and characterization of its weathering processes are essential steps in the scientific approach to the preservation of wooden cultural material, Although numerous priceless wooden artifacts from ancient Egypt can be found in museums, there is comparatively little knowledge about the type of wood used and how well they are being preserved. Three important archaeological wood objects a statue, a box, and a coffin found at various Egyptian archaeological sites and dating from the Old Kingdom (2,686–2,181 BC) to the New Kingdom (1,550–1,069 BC) were thoroughly studied to fill this knowledge gap. Five species of hardwood and softwood, including *Tamarix manner*, *T. gennessarensis*, *Ficus sycomorus*, *Vachellia nilotica*, and *Cedrus* sp. were identified. Microcrack development, biological deterioration patterns (fungal colonization) indicated the existence of fungal hyphae and conidial spores on the wooden objects.

Microorganisms that congregate on wooden shipwrecks and use the matrix of the wood to develop and multiply can cause bio-corrosion and biodegradation when an ancient shipwreck is exposed to the air. *Acinetobacter* was the most prevalent bacterial genus, breaking down lignin and cellulose whereas *Penicillium*, *Aspergillus* and *Cerrena* were the most prevalent fungus genus destroying lignin, providing a reference point for the preservation of the ship in the future (Ma et al., 2022). The primary degraders of prehistoric wood discovered in damp situations are erosion bacteria. Chemical investigation proved that it significantly depletes holocellulose causing enzymatic unlocking of the lignocellulose to get access to the holocellulose portion of the cell wall leading to chemical alterations in the lignin polymer (Pedersen et al., 2021). Appiah-Kubi et al. (2021) recorded the preservation of wood and restoration of artifacts against wood-destructive organisms. Additionally, Broda and Hill (2021) noted the need to conserve waterlogged timber by utilizing some modern, environmentally friendly preservatives and effectively protecting old timber objects (Broda et al., 2021).

The susceptibility to biodegradation of ancient wooden artifacts could be eliminated by using some modern, bio-friendly preservatives, offering effective protection. Essential oils have been used in conservation approaches that are harmless for people and the environment, for cultural civilization, effective against a broad range of microorganisms and capable to be used in remote regions. This has allowed us to accept their use in replacing artificial biocides in the environmental conservation of cultural objects. *Thymus vulgaris* L. (Lamiaceae) essential oil and hydro-alcoholic were applied to stop microbial foundation (*Aspergillus* sp., *Streptomyces* sp. and *Micrococcus* sp.) on wooden sculpture surfaces. The plant’s bioactive materials are extracted as solutions and a synergistic influence of *T. vulgaris* extracts (essential oil and hydro-alcoholic solutions) was proposed for a particular wooden statue. The idea was that after alcohol loss, the *T. vulgaris* antimicrobial substance should remain on the sculpture’s surface, improving the antimicrobial activity of volatile composites. This is a further applicative procedure, respectful of both workers and the ecosystem, replacing synthetic biocides in the supportable preservation of cultural resources (D’Agostino et al., 2021; Sparacello et al., 2021). Palla et al. (2021) used the plant essential oils in supervisory fungal colonization on lacquerware wooden objects. The fungal strains were isolated and then recognized by the magnification of ITS-18S rRNA. *Penicillium chrysogenum* (NK-NH3) and *Fusarium solani* (NK-NH1) were the principal. Four biocidal products repressed the growth of the fungal types *in vitro* efficiently.

**Figure 2:**
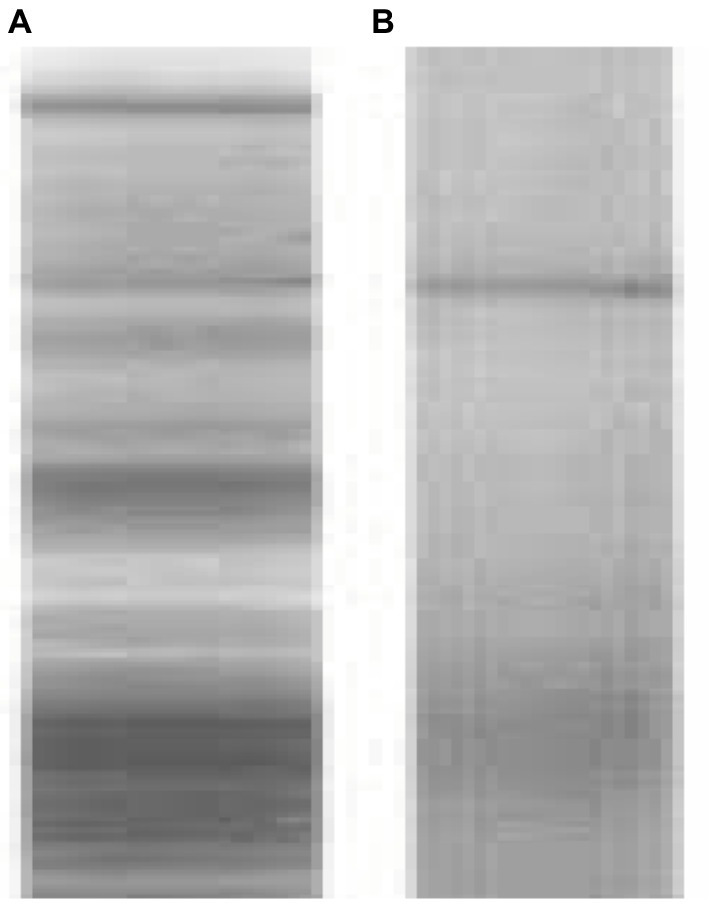
Non laser irradiated protein of *Cladosporium cladosporioides* Lane **(A)** Laser irradiated protein of *C. cladosporioides* showing microbial inhibition by disappearance of all major protein bands Lane **(B)** (Geweely, 2006).

Laser is a convenient, selective and contactless technique, which does not announce any damaging chemicals to people, environment and the culture material. However, they are expensive, not selective and have restrictions on the application in remote regions. It has been used for the management of *A. flavus* in marble and was positively used as fungal inhibition. Assessments for optimization of laser issues were performed developing in a 100% decline in the number of *Aspergillus* spores (Becerra et al., 2020; Palla, 2020; Palla et al., 2020; Parfenov, 2020; Rybitwa et al., 2020; Díaz-Alonso et al., 2021; Romero et al., 2021). The great energy transmission involved by laser radiation breaks DNA chains and protein links, which affects the loss of all major protein bands as shown in Figure 2.

Omar et al. (2022) discovered that the wood included in an old boat, which belonged to Khufu, the second ruler of Egypt’s Fourth Dynasty, was decayed by microorganisms. As fungi and bacteria quickly attack and metabolize wood due to its typical sensitivity to biological attacks, physical, chemical, and morphological changes. *A. niger*, *A. flavus*, *A. sulphureus*, *Penicillium janthinellum*, *Cladosporium herbarum*, *Botryotrichum piluliferum* and *Bacillus megaterium* were the responsible microbes. Pentachlorophenol at 900 ppm is the optimal concentration of a particular microbicide for the bio-treatment of contaminated wood materials since it is sufficient to inhibit all isolated microorganisms. Jia et al. (2020) recorded that the ITS-18S rRNA gene was used to identify the isolated fungus from old objects as *P. chrysogenum* (NK-NH3) and *Fusarium solani* (NK-NH1). The predominant fungus was *Fusarium*, which might have been introduced by tourists or conservation restorers from the outside environment. The biocide susceptibility assay revealed that isothiazolinones efficiently suppress the growth of fungal isolates. Lindane, pentachlorophenol, alkaline chloride, sodium chloride, fluorosilicates were found to be reliable and effective materials for preserving and restoring wood and wood-based products. These methods included fine and coarse spraying, brushing, smoking, soaking or dipping, impregnation, injection, and infusion (Appiah-Kubi et al., 2021).

### 2.3. Conservation of animal origin organic microbial deteriorated archaeological objects

#### 2.3.1. Painting and parchment

All paintings are crucial elements of cultural heritage, they are made of organic compounds (oils, waxes, gums, sugars, polysaccharides and proteins) that can help a variety of microbes flourish and could be used to develop a plan to reduce the number of visitors and manage microorganisms to halt the biodegradation process (Suphaphimol et al., 2022). Ancient wall paintings may experience esthetic modification as well as structural harm from microbial decay. The main bacterial genera were *Pseudonocardia* and *Streptomyces*, while the main pathogenic fungi belonged to the Ascomycota phylum. Dead insect remains and organic adhesive ingredients used to create the wall paintings serve as vital nutrients that encourage the rapid growth of bacteria and saprophytic fungi on the wall paintings. The cave door should be opened more frequently according to the weather to prevent the interior from being too humid and also the exogenous nutrients such as dead insects inside the cave are cleaned (Ding et al., 2020).

The dark blotches in the nearly 1700-year-old underground ancient tombs are due to the microbial decay of the brick mural paintings. There were 22 isolated fungi, all of which belonged to the *Penicillium* and *Aspergillus* genera. More than 68% of the isolated fungus exhibited proteolytic activity and 27% of the strains generated acids that caused calcium carbonate to dissolve, so brick wall paintings are under serious risk by the flourishing fungi (Ma et al., 2022). *Aspergillus*, *Penicillium*, *Cladosporium*, *Alternaria*, *Curvularia*, *Chaetomium* and *Trametes* were the seven dominant genera of detected fungi. The *Bacillus*, *Staphylococcus*, *Micrococcus*, *Paenibacillus*, *Arthrobacter*, *Heyndrickxia*, *Priestia* and *Rathayibacter* were isolated bacteria (Noohi and Papizadeh, 2022). In the site with the most visitors to the painting in Thailand temples, *Aspergillus* was the most prevalent genus among the different fungi communities. While the *Neodevriesia* genera dominated the field of mural painting. The most common type of bacteria was gammaproteobacteria. The number of visitors was connected to human-caused microbial pollution, while the percentage of saprotrophs in the local microbiome was higher in the temple (Suphaphimol et al., 2022). On wall paintings kept in a Chinese museum, eight fungus taxa were found, where *Cladosporium*, *Penicillium*, *Alternaria* and *Filobasidium* being the most prevalent.

The spread of airborne fungi is influenced by relative humidity, temperature, and seasonal precipitation. The details for the warning conservation of cultural artifacts are kept at nearby locations and in museums (Duan et al., 2021), where the use of biocides for urgent wall painting conservation and the development of a preventative protection system based on micro-environmental control will provide technical assistance for the long-term preservation of historic wall paintings (Dongpeng et al., 2021). *A. niger*, *A. flavus* and *Alternaria alternata* have already damaged the mural painting surfaces of the tomb. The isolation of three species of *Trichoderma* (*Trichoderma harzianum*, *T. hamatum* and *T. aureoviride*) were recorded as biocontrol agents. The optimal conditions for enhancing *Trichoderma* spp. bioactivities were 5% sodium nitrate and sodium chloride crystallization in the tomb, average temperatures between 30 and 35°C, and an acidic pH of 5.5.

The safe and clean method for creating nanoparticles (NPs) as green chemistry is seen to be an appropriate strategy for the environmentally friendly creation of ancient parchment. Tea tree leaf extract was utilized to create environmentally safe silver nanoparticles (Ag-NPs). It’s interesting to note that some bacteria and fungi can produce metabolites that are used to create various nanoparticles. These NPs have interesting physicochemical characteristics, such as an ultra-small size, a high surface-to-mass ratio, and a peculiar reactivity with organisms, making them useful for both organic and inorganic materials. Some nanoparticles (NPs) made of zinc oxide (ZnO), copper (Cu), titanium dioxide (TiO_2_), or silver (Ag) exhibit intriguing biocidal properties against degrading bacteria. The environment and heritage materials are continually threatened by biocidal treatments since they are short-lived and regularly repeated (Wawrzyk et al., 2020). The microbiostatic effect of the green synthesis Ag-NPs disinfection process was attained at a concentration of 0.005%, while the microbicide effect was attained at a concentration of 0.025% for the deteriorating microorganisms (*Aspergillus fumigatus*, *Byssochlamys spectabilis*, and *Streptomyces albidoflavus*) that isolated from historical parchment. Disinfected parchment’s chemical and mechanical qualities had no discernible impact. Ag-NPs may therefore be a viable option for the long-term protection of historical parchment from microbial biodegradation (Saada et al., 2020). New technologies must be used to diagnose, treat and safeguard wall paintings and murals, including the use of omics technologies for diagnosis and nanoparticles for treatment (Wu et al., 2022).

The most efficient way to apply essential oils would be to flow a thin layer of it into a surface that was already evaporating and place it close to the painting using some supports so that the essential oils’ vapors could reach the painting’s surface uniformly while avoiding direct contact between the EOs and the pigments (Gatti et al., 2020). On two antique Indian leather puppets, D’Agostino et al. (2021) applied the essential oils of *Thymus vulgaris* L. and *Crithmum maritimum* L. as a biocide. The essential oils function better against microbes when prepared as nanoemulsions, which makes it possible to achieve biocidal action at lower concentrations without negatively impacting any of the properties of the disinfected parchment. However, applying the oil in its regular form had an impact on the optical characteristics of the artifacts, so historical parchment can be properly disinfected using essential oils nanoemulsions, which are thought of as an eco-friendly technique, without suffering any negative effects on their distinguishing qualities (Saada et al., 2020). On a canvas painting, 3% concentration of liquorice leaf extract was evaluated for the removal of mixed patinas, which included bacterial species *Bacillus licheniformis* and *B. subtilis* as well as fungus species *Arthrinium* sp., *Aspergillus* sp. and *Cladosporium* sp. The leaf extract was efficient in both lowering the microbial load and stopping the re-proliferation of germs over time (up to a year later) (Sprocati et al., 2021). The essential oil was employed by Palla et al. (2020) as natural biocides in the preservation of cultural assets. The biocidal ability of two plant derivatives, as the essential oils of oregano and cloves were assessed as a potentially new and environmentally friendly cleaning and conservation technique for canvas paintings. The fungus genera *Penicillium*, *Aspergillus*, and *Cephaloteca* as well as the genus *Bacillus bacterium* were the dominant colonizers of the canvas.

Green natural biocide was achieved by the growth of *Trichoderma* species as antifungal agents in the tomb and was used against *Alternaria alternate*, *A. niger* and *A. flavus. Trichoderma* species can be used to regulate the deterioration of cultural assets. It is a risk-free, environmentally acceptable method that can be used anywhere, *in vivo* or *in vitro*, in cultural heritage open doors or museums (ElHagrassy, 2022). Interestingly, *Bacillus*-based therapies have been proposed to protect cultural heritage items against fungi (Silva et al., 2017; Caselli et al., 2018). Jurado et al. (2020) recommended the use of microbial by-products (acids, extracellular enzymes) or bacterial extract, complete microorganisms, plant extract and essential oils against degrading microorganisms. Also, Catto et al. (2020) suggested the bio-cleaning of organic graffiti using innovative commercial strains of bacteria. Enzymes can therefore be viewed as practical resources and reliable operating procedures for practitioners in various conservation fields. The advancement of enzyme usage in the conservation of art was found by Cremonesia and Casoli (2021). The critical and historical perspective of scientific studies is provided by the use of microorganisms and enzymes in the bio-cleaning of cultural heritage artworks (Ranalli and Zanardini, 2021).

On the other hand, Nabil et al. (2021) claimed that mechanical cleaning is a crucial step before microbial treatment because it reduces microbial load and consequently, decreases the dose of antimicrobial needed for treatment. Additionally, the mechanical cleaning process offered a secure method to be used in fabric conservation. Also, chemical treatment is required to get rid of microbiological issues.

Traditional chemical products like benzalkonium chloride, o-phenyl phenol, and tributyltin naphthenate can be combined with natural varnishes to preserve artwork against environmental fungus and bacteria without affecting the materials’ inherent properties or the way the pieces look (Romero-Noguera et al., 2020). Eldeeb et al. (2022) used dry cleaning and disinfection as conservation techniques for the most significant photographic prints from the late 19th century are albumen prints which consist primarily of two layers: the paper support (cellulose) on the top and the image layer on the bottom (image silver particles embedded in an albumen binder layer).

#### 2.3.2. Mummies

Scientists can reconstruct the evolution and appearance of past diseases as well as the customs and lifestyles of ancient societies acknowledges to mummy investigations (Bianucci et al., 2022b). Mummies are a vital and valuable component of Egyptian and world cultural heritage. They might be viewed as intricate artifacts constructed primarily of linen, salt, essential oils, and mummified materials. Xerophiles (osmophiles) grow on dry meat and halophilic fungi can also handle high salinity, the range of materials utilized and environmental variables have a considerable impact on their infection by xerophiles and halophilic fungi that work together for mummy deterioration. Therefore, conservation techniques must be created effectively to maintain the remains without diminishing their significance as a source of biological and scientific information (Ali et al., 2022).

The mummy was significantly affected by the humidity because the water molecules’ reaction with the mummification salts created a fundamental media that aided the fungi’s growth. The Natron salt and the water molecules of humidity reacted to create a basic media and the humid environment encouraged microbial development (Magdy et al., 2020). The study of human mummy samples revealed contamination by halophilic bacteria to several mummies and had highly mold-contaminated surfaces (Bianucci et al., 2022a). The chemical, physical, biological and environmental origins are crucial for the proper conservation of ancient remnants (Mustieles et al., 2021). Humidity and aeration are the two more important elements that may stop certain strains from growing. The taxa *Aspergillus* sp. and *Chaetomium* sp. of a filamentous fungus, as well as *Paenibacillus* sp., *Staphylococcus* sp., *Staphylococcus* sp. and *Staphylococcus epidermidis*, were isolated from ancient mummy. Analysis of the chemical and surgical procedures used on cadavers in mummies from the 16th to the 20th centuries was detected by Bianucci et al. (2022b). Cellulase, amylase and protease activities were used to assess the possible bio-degradative microbiota present in samples recovered from two Spanish mummies from the 18th and 13th centuries. By using the PCR technique, *Mycobacterium tuberculosis*-specific gene was the potential existence pathogen (Mustieles et al., 2021). DNA was used to examine the microbiological deterioration of archaeological bone by bacteria and fungus, which gives information about the presence rather than the activity of incidental taxa (taxa that are present but not actively destroying bone) (Emmons et al., 2020).

Geweely et al. (2023) stated that mummies are a fundamental and important part of both the Egyptian and the global cultural heritage. The variety of materials used and environmental factors have an impact on how susceptible it is to fungal colony infestation. Mummy deterioration due to microbial activity is a widespread issue, keeping it clean over time and safe for future generations is difficult. One of the main elements that significantly contribute to mummy destruction is fungus deterioration. New chalcone derivatives were created, and their antifungal properties were tested *in vitro* against 13 isolated deteriorating fungal species (*A. flavus*, *A. niger*, *A. terreus*, *Athelia bombacina*, *Aureobasidium iranianum*, *Byssochlamys spectabilis*, *Cladosporium cladosporioides*, *C. ramotenellum*, *Penicillium crusto-sum*, *P. polonicum*, *Talaromyces atroroseus*, *T. minioluteus* and *T. purpureogenus*) isolated from Egypt’s ancient mummy. The bulk of the isolated core phyla was Ascomycota (*A. flavus*, *Aspergillus terreus*, and *A. niger*). The most effective novel chalcone derivative with three methoxy groups acting as an electron-donating group and one methoxy group acting as an electron-withdrawing group applied at a minimum inhibitory concentration (MIC) of 1 to 3 mg/ml instead of using physical and chemical disinfection to prevent adverse effects on the artwork, environment and public health. Also, Ali et al. (2022) documented a conservation strategy for the preservation of multi-piece mummy cartonnage from the Late Period (780 BC–332 BC). The enzymatic synergistic bacterial action of *Micrococcus* sp. and *Microbacterium* sp. strains appears to be the source of the mummy’s biodeterioration. A remarkable early therapy using gamma radiation was given to Ramses II’s mummy, which was being attacked biologically and suffering from stains, particularly from fungi. To prevent contamination, the mummy has been irradiated and placed in a sterile case in a museum (Cortella et al., 2020). Using ribosomal ribonucleic acid (rRNA) analysis, *Bacillus jeotgali*, *Kocuria turfanensis*, *Microbacterium imperial*, *Micrococcus luteus*, and *Bacillus megaterium* were isolated from the degraded mummy and were inhibited by three types of microbicides nanomaterials (zinc oxide), plant extraction (*Ceratophyllum demersum*) and chemical materials (4-chloro-m). The most effective antibacterial agent is *Ceratophyllum demersum*, a plant extract, at a concentration of 600 ppm/100 ml, where it is adequate to prevent the growth of all isolated bacteria (Ismael et al., 2021).

## 3. Conservation of inorganic deteriorated archaeological objects

### 3.1. Stone

A historical building is characterized as one or more structures containing a variety of artifacts that need to be continuously preserved to maintain their historical architectural esthetic and cultural significance. Each ornamental room in the building serves as a cue to jog memory because each piece has a distinct mental orientation (Hasan, 2023). One of the research areas preservation of historic structures and monuments is stone degradation. The metabolic events that take place in the carbon, nitrogen and sulfur cycles are primarily responsible for the microbial degradation of inorganic materials. The availability of water, the presence of microorganisms, the movement of soluble salts during wet and dry cycles, and other material porosity properties all have a big impact on how durable stone materials were degraded. The most crucial component before microbial colonization and subsequent biodeterioration processes begin is the water linked with cultural heritage items (Li and Gu, 2022).

The primary problem for microbiologists is the removal of bio-deteriorated microorganisms from stone (De Leo and Jurado, 2021). Physical, chemical, and microbiological elements have all been formally linked to stone biodeterioration. Numerous studies have documented the colonization of stone by microorganisms, as well as the dissolution and loss of CaCO_3_ in the stone’s deterioration over time from exposure. This increases the stone’s porosity to trap atmospheric depositions and promotes the development and growth of microorganisms due to the stone’s improved ability to hold water and the availability of nutrients. There are a direct relationship between mineral dissolution reactions and microbially catalyzed contributions to them (Qian et al., 2022). Also, NO_2_ and SO_2_, feed nitrifying bacteria (*Nitrosomonas* and *Nitrobacter*) and sulfur-oxidizing bacteria (*Thiobacillus*). The microbial weathering phenomena toward limestone were studied by Ahmed and Mohamed (2022), while the microbial biodeterioration processes affecting the sandstone cultural heritage will help in the protection and management of the ancient temple was reported by Ding et al. (2020). *Rubrobacter*, *Arthrobacter*, *Roseomonas* and *Marinobacter* taxa are thought to be responsible for the creation of colored biofilms, while *Ulocladium, Cladosporium* and *Dirin*a connected to structural damage. The most significant detergents are *Bryobacter*, *Chroococcidiopsis*, *Rubrobacter*, *Blastocatella*, *Sphingomonas* and *Loriellopsis*, where the investigation of phototrophic biofilms revealed the existence of many predators operating naturally to regulate the growth of photosynthetic-based biofilms in caves (De Leo and Jurado, 2021). Kosznik-Kwasnicka et al. (2022) investigated the caves and discovered a wide variety of bacteria, algae, and fungi dwelling on stone walls and develop inside the rock, where black fungi are one of the most threatening risks to the stone cultural heritage of the Mediterranean basin. The black fungus’s capacity to generate a chemical effect on carbonate stones and affect other materials/historical artifacts by the creation of acid, cellulase, esterase, and protease, so the possibility of using them as reference organisms were made by the achievement of their sensitivity to four conventional biocides (Isola et al., 2022). To improve the conservation of stone biodeterioration for protective management, it is crucial to identify the key microbial biochemical events. The number of genes related to acid tolerance and chemotaxis increased in bacteria living in granite, while bacteria living in limestone have more genes related to photosynthesis and radiation resistance (Brewer and Fierer, 2018). The variety of eukaryotes considerably differed with different geographic locations rather than seasons and the diversity of prokaryotes in the archaeological limestone showed considerable temporal and regional changes (Wang et al., 2022).

The risk management and conservation of historic structures museum were investigated by Tuba and Dişli (2022) who stated that the diversity, distributions, ecological roles, and interaction patterns of the fungal and microalgal (including cyanobacteria and algae) communities on sandstone in Temples were obtained using high-throughput sequencing analysis. The core phyla of fungi were affiliated with Ascomycota (Wu et al., 2022). There are two strategies to combat biodeteriogens, according to Mascalchi et al. (2020), who applied biocides against the target microorganisms on stone, being safe for the treated item, being simple to remove, and not showing any chemical or esthetic interference with the stone surface after cleaning. The current conservation standards state that following cleaning, preventative measures must be developed to prevent a new recolonization and/or temporarily limit the growth rate of biofilms.

The use of essential oils for disinfection is aesthetically acceptable and has low toxicity for humans and the environment, ensuring a high standard of living for workers and users and preserving cultural heritage. The development of environmentally friendly biocides against microbial deteriorated tombs, where opening, flooding, upper vegetation, visitor entrance and conservation treatments play a significant influence in the biodeterioration processes (Caneva et al., 2020a). Conventional cleaning techniques with controllable, selective, contactless, and ecologically friendly natural biocides have been studied on outdoor stone surfaces (Barreiro et al., 2020). Also, due to its advantages over conventional cleaning procedures, where it is controlled, selective, contactless, and ecologically benign, nano-encapsulated essential oils have been studied on outdoor stone surfaces by Romano et al. (2020). Lavender essential oil and liquorice leaf extract were employed as plant derivative biocides to combat phototrophic biofilm that was developing on stones (Rugnini et al., 2020). Environmentally friendly stone conservation treatments are moving toward low-impact biocides such as plant natural extracts to combat microbial deterioration. To create two separate combinations for the treatment of the statue, four essential oils (*Coridothymus capitatus*, *Syzigium aromaticum*, *Cinnamomum zeylanicum*, and *Origanum vulgare*) were selected and combined. Because of the low concentrations employed, there is no environmental bioaccumulation and no risk to humans. Additionally, the statute will be continuously observed to document the long-term effects of the applied therapies (Spada et al., 2021). The application of a bio-cleaning procedure on granite, the potentiality of natural biological control of phototrophic biofilm in caves, the biocidal activity of natural products derived from plants, and other green treatment methods are effective but not harmful to the material, operator, or environment (Sparacello et al., 2021). Evaluation of the impact of the principal fungus and bacteria causing the biodeterioration of gypsum work using essential oils (thyme, clove, cinnamon, garlic, castor, and olive) was evaluated by Khali et al. (2022), where *Aspergillus japonicas*, *A. terrus*, *Penicillium commune* and *Cladosporium elatum* were the four fungal isolates, while *Bacillus cereus* and *Listeria monocytogenes* were the two bacterial species. The most efficient natural product for preventing the biodeterioration of Gypsum archaeological work was garlic oil, which had the best effects on all isolates.

A unique comparison of chemical, natural essential oil, and physical (ozone) for preservation of archaeological items against microbial deterioration was documented by Geweely et al. (2022). Chemical preservation of historical artifacts poses a risk to both the environment and human health, as well as degradation (erosion and surface damage). Microbial burdens can be successfully reduced by ozonation, where ozone may replace chemical sanitizers as a common sanitizing agent due to its strong oxidizing capacity and spontaneous disintegration.

The effectiveness of mixing eugenol with an environmentally friendly emulsifier (Phyto-derivatives) as new biocides, such as the potential of allelopathic compounds generated from lichens, appear to be promising in the field of cultural heritage conservation (Caneva et al., 2020b). Jurado et al. (2020) showed that phototrophic microbes (*Bacillus* and *Lysobacter*) on lithic substrates (monuments and walls) in caves, may be controlled using a new technique that combines chemical and biologically generated biocides. In China, sculptures were damaged by lichen and fungi, and an efficient conservation technique was investigated by three different antimicrobial medicines were evaluated for 2 years to prevent microbial assault. The most effective combination was biocide and water repellent (Wang et al., 2021). Additionally, the combination of a conventional chemical substance with natural varnishes, such as tributyltin naphthenate, o-phenyl phenol, and benzalkonium chloride, should help shield polychrome sculptures from environmental fungi and bacteria without affecting the original materials or the visual appeal of the artworks (Romero-Noguera et al., 2020).

The majority of the tomb murals that have been kept at their original locations are at risk from microbial deterioration, and the long-term management of these microorganisms is a perennial issue in the field of cultural asset conservation. Numerous mycelia with conidia were present, and the culturable fungi in the white mycelium samples belonged to the six genera of the Ascomycota phylum. Dichlorophene compounds (0.5% dichlorophene with 75% ethanol) were the most effective biocide. Throughout the 7 years of continuous monitoring, no recurrent outbreaks of microorganisms. To achieve long-term prevention to the tomb, it is advised to combine emergency protection, environmental regulation, and follow-up monitoring in the future (Fasi et al., 2022). *B. cereus* OK447647, *B. subtilis* OK447648, *Serratia marcescens* OK447650, *Pseudomonas oryzihabitans* OK447649, *A. flavus*, *A. niger*, *P. chrysogenum* and *Cladosporium cladosporoids* were the most representative bacteria and fungi that were isolated from the building’s air and limestone indoors and outdoors. The optimum treatment for bacterial isolates was found to be sodium azide at 100 ppm, although it had no discernible impact on fungi. Additionally, it is important to monitor and maintain anthropogenic and environmental influences within a range that is optimal for historic stone monuments while offering protection from microbial colonization (Aydın et al., 2022). Ionic liquids technologies, which can help produce new formulations of antifouling and antimicrobial surface coatings, are one area of research that Lo Schiavo et al. (2020) recorded as a way to combat the proliferation of microbes and the formation of biofilm on stone monuments.

## 4. Conclusion

The present study reveals that the effective conservation methods for each unique cultural heritage piece consequently should be on a case-by-case base based on scientific evidence. Interdisciplinary methodologies are required for effective preservation techniques combined with microbiologists, geologists, chemists, ecologists, archeologists and physicists. The Ascomycota phylum contained the majority of the pathogenic flora, which are problematic to destroy or eliminate due to their capability to flourish inside the artifacts is represented by *Aspergillus* which was a dominant species of black fungus that can survive inside an object and resist a range of stresses. Both human allergies and microbial contamination from humans were associated with a high visitor count, and this information might be applied to setting a strategy to diminish the number of visitors. To develop a science-based protection strategy monitoring microbial degradation including the use of omics technologies for diagnosis, environmental conditions (continuous airflow, ventilation, photocatalytic ionizers, temperature), anthropogenic effects and knowledge of the mural materials structure. New biological technologies (green biocides) represent a promising recent strategy to protect cultural heritage from biodeterioration as save eco-sustainable alternatives harmless to humans and the environment, environmentally friendly, safe, ecologically acceptable alternatives stop microbial deterioration, assess the perseverance of treatment on surfaces over time with a short-term and long-term investigation, evaluate the costs, avoid any possible interactions between the biological agent and the artifacts. Also, a synergistic effect of combining natural biocides with mechanical cleaning or chemical treatment was suggested recently.

## Author contributions

NG: the idea of manuscript, manuscript writing, and data interpretation.

## Conflict of interest

The author declares that the research was conducted in the absence of any commercial or financial relationships that could be construed as a potential conflict of interest.

## Publisher’s note

All claims expressed in this article are solely those of the authors and do not necessarily represent those of their affiliated organizations, or those of the publisher, the editors and the reviewers. Any product that may be evaluated in this article, or claim that may be made by its manufacturer, is not guaranteed or endorsed by the publisher.
